# Supplementary material for: Dynamic and rapid deep synthesis of chemical exchange saturation transfer and semisolid magnetization transfer MRI signals
Source: Sci Rep. 2023 Oct 25;13:18291. doi: 10.1038/s41598-023-45548-8 (PMC10600114; doi:10.1038/s41598-023-45548-8)
Supplement: Supplementary file 1 — Supplementary Information. [file 41598_2023_45548_MOESM1_ESM.pdf]

Supplementary Information: Dynamic and Rapid Deep Synthesis of Chemical Exchange Saturation Transfer and Semisolid Magnetization Transfer MRI Signals

Dinor Nagar[1], Nikita Vladimirov[2], Christian T. Farrar[3], Or Perlman[2,4*]

[1] *School of Electrical Engineering, Tel Aviv University, Tel Aviv, Israel*

[2] *Department of Biomedical Engineering, Tel Aviv University, Tel Aviv, Israel*

[3] *Athinoula A. Martinos Center for Biomedical Imaging, Department of Radiology, Massachusetts General Hospital and Harvard Medical School, Charlestown, MA, USA*

[4] *Sagol School of Neuroscience, Tel Aviv University, Tel Aviv, Israel*

[*] Correspondence to: Or Perlman, Department of Biomedical Engineering and Sagol School of Neuroscience, Tel Aviv University, Tel Aviv 6997801, Israel. Email: orperlman@tauex.tau.ac.il

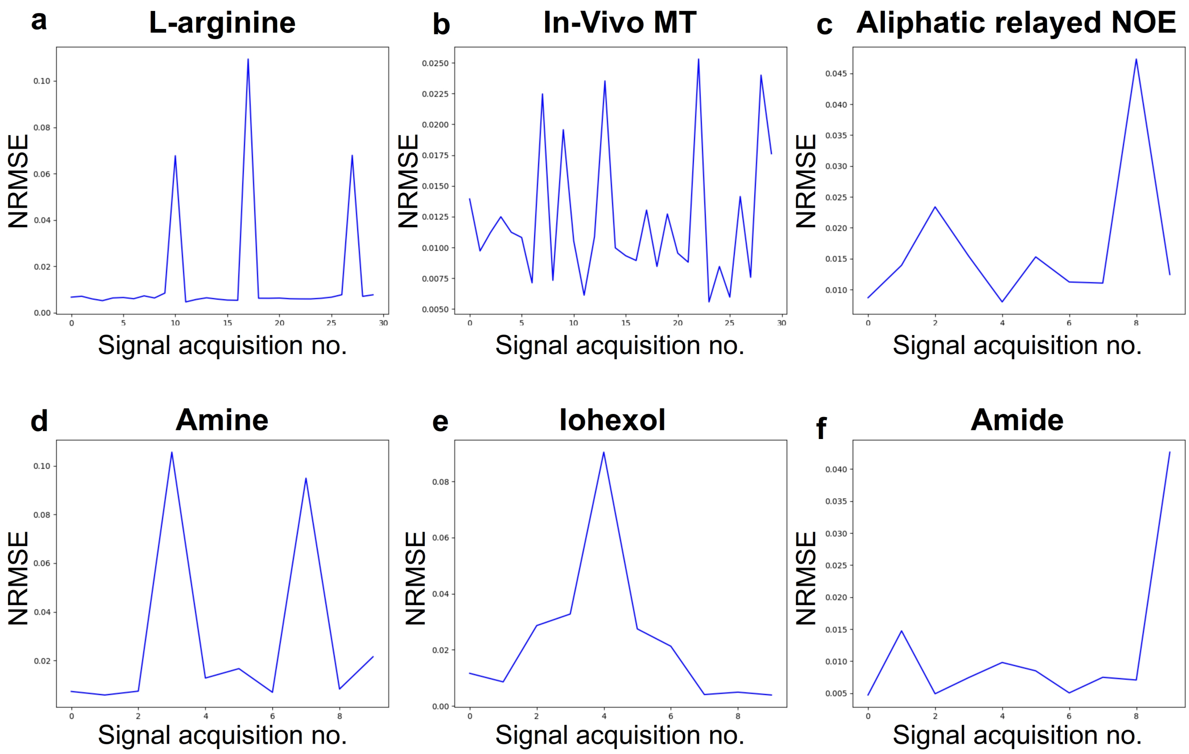

Supplementary Information Fig. S1. Normalized root mean square error (NRMSE) dependence on the signal evolution step (s_i), for each of the imaging scenarios shown in Fig. 2.

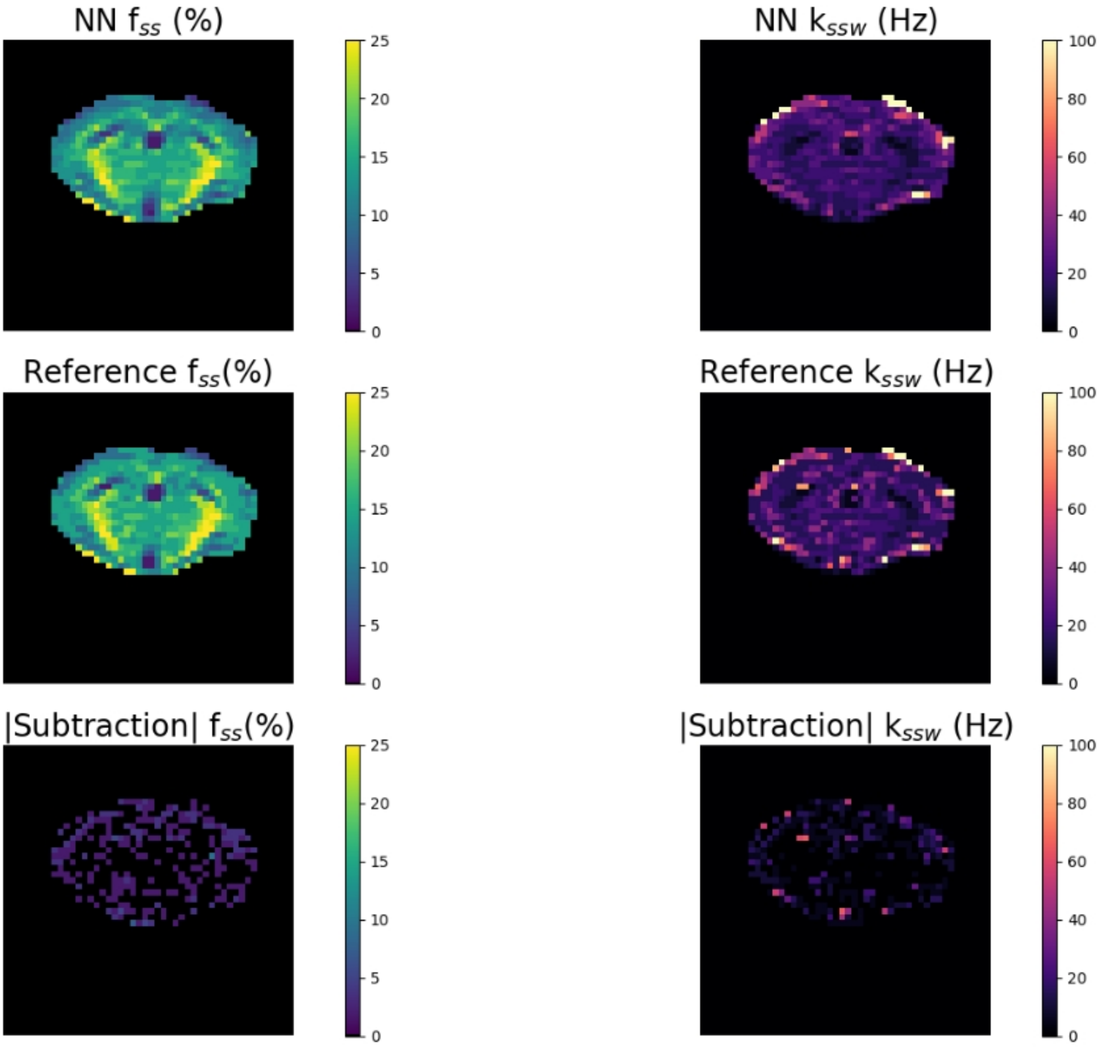

Supplementary Information Fig. S2. Magnetic resonance fingerprinting quantitative reconstruction of in-vivo semisolid MT proton volume fraction (**left**) and exchange rate maps (**right**) from a wild-type mouse. The images were reconstructed using dynamic-NN-based signals (**top**) and traditional numerical solution of the BM equations (**center**). The absolute difference images between the NN-generated and the ground truth reference are presented at the last (**bottom)** row.

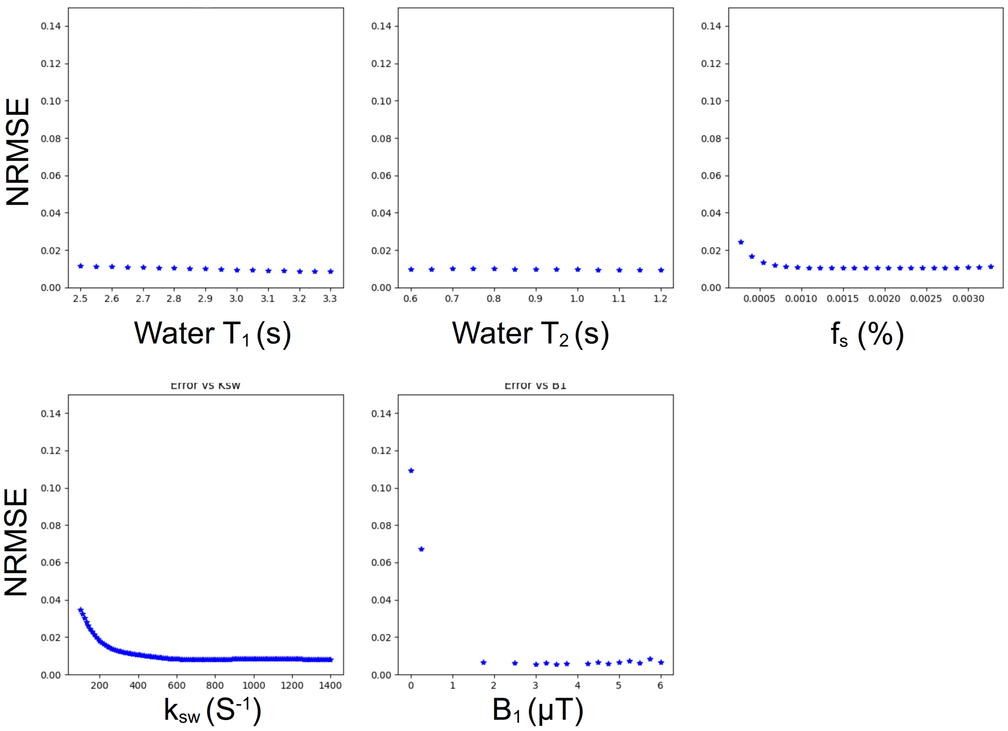

Supplementary Information Fig. S3. Dynamic network performance with respect to tissue and acquisition parameters. The normalized mean squared error (NRMSE) were calculated as a function of water relaxivity (T_1/T_2), proton volume fraction (f_s) and exchange rate (k_{sw}), and the saturation pulse power (B_1), for a representative imaging scenario (L-arginine, see Fig. 2 and Fig. 3).

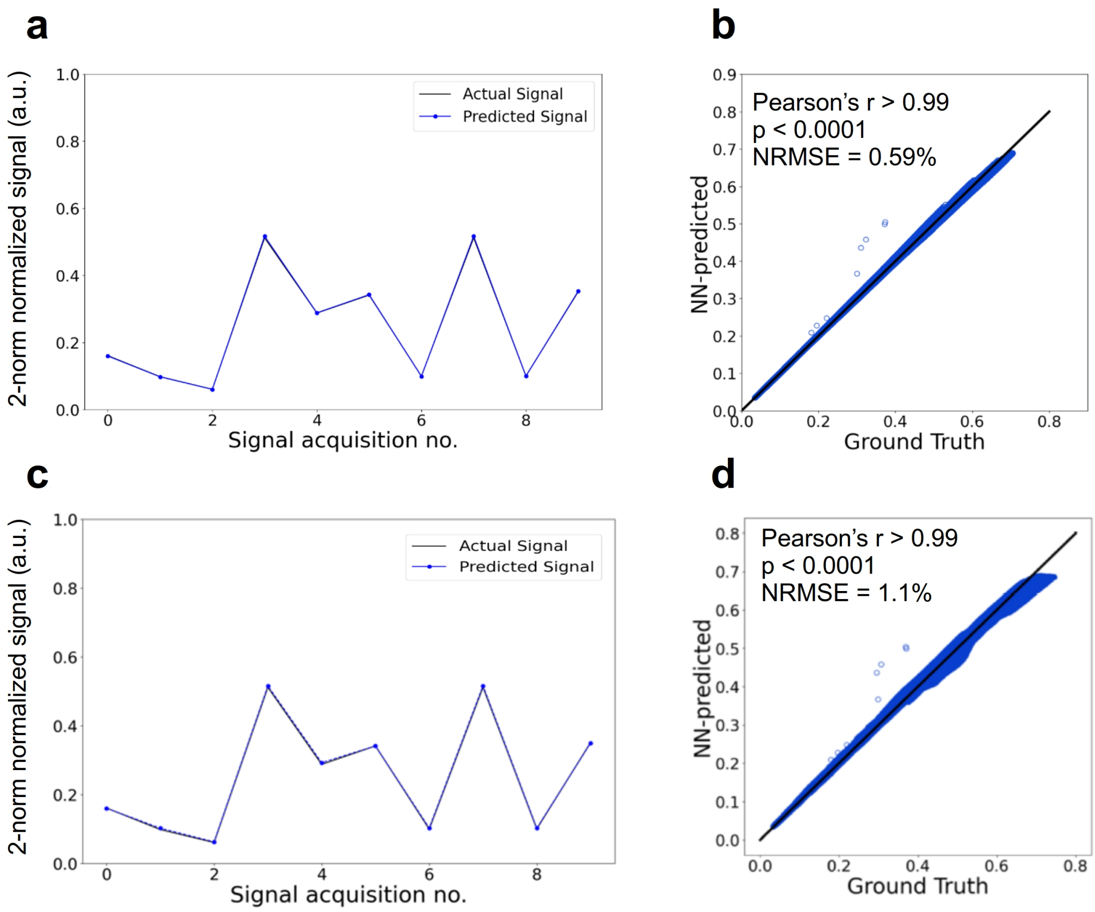

Supplementary Information Fig. S4. A comparison between the application optimized (top) and adaptive (bottom) architecture performance, for a representative imaging scenario (a two pool amine CEST MRF acquisition). **a, c.** A representative simulated signal. **b,d.** Statistical analysis of the agreement between the ground-truth signals and the NN-simulated signals using both architectures.

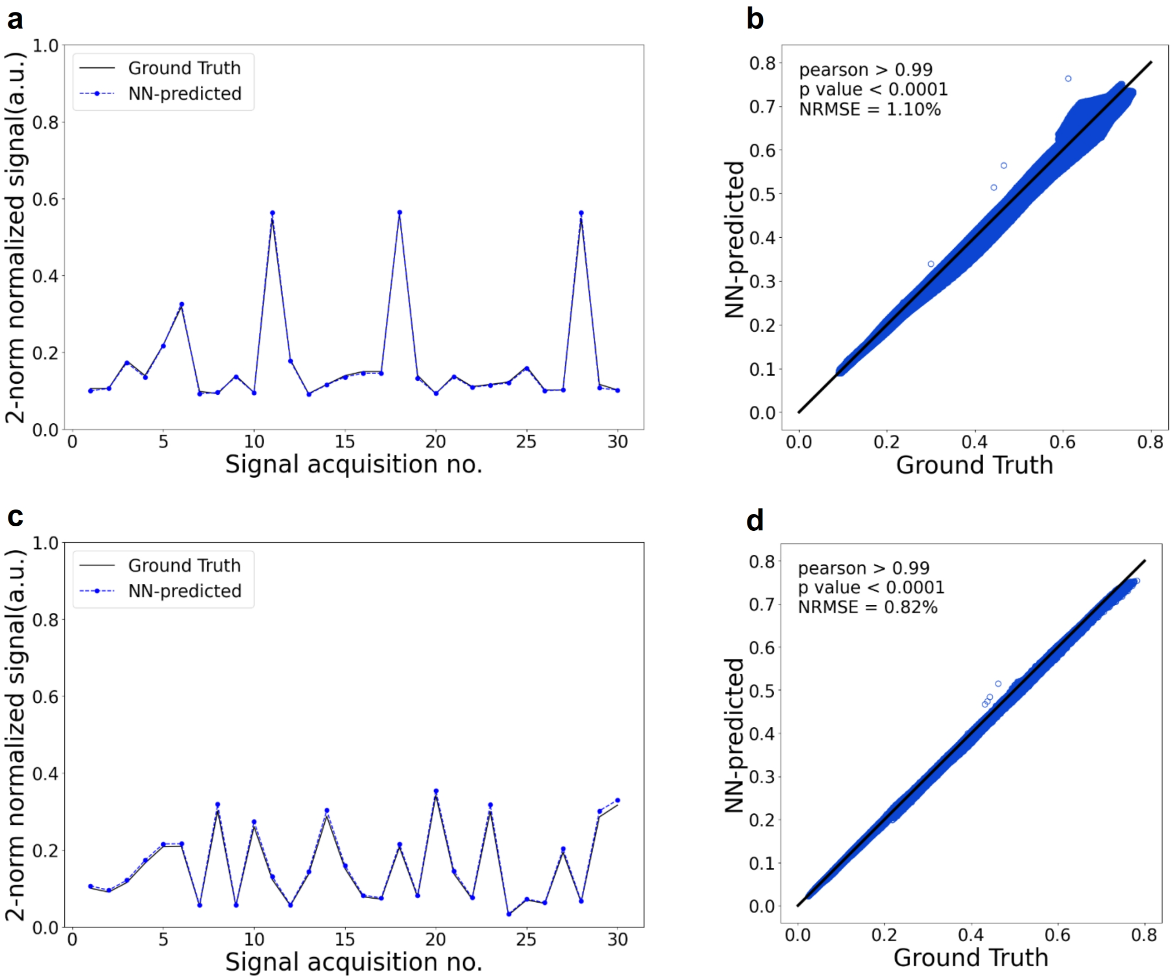

Supplementary Information Fig. S5. In-silico comparison between dynamic NN-generated CEST (top) and semisolid MT (bottom) signals at 7T and their ground-truth counterparts. (**a**, **c**) Representative test-set signal trajectories and full statistical analysis (**b**, **d**) for the L-arginine (top) and semisolid MT imaging (bottom) scenarios[34], respectively, generated using the same single dynamic NN used in Fig. 2 and Fig. 3. Note the excellent agreement (Pearson's r>0.99, p<0.0001) between the NN-generated trajectories and the ground-truth reference.

Supplementary Information Table 1. Comparison between the different acquisition protocols and their signal creation time using the NN or reference ground truth framework.

Imaging scenario	Number of entries	Reference synthesis using a 12-core CPU (sec)	Neural network using a 12-core CPU (sec)	Neural network using 3060TI GPU (sec)
**rNOE**	176,800	19.18	2.08	1.08
**Amine**	397,800	23.43	4.31	1.42
**Amide**	286,520	21.08	3.17	1.18
**MT**	26,400	20.76	1.70	1.60
**L-arginine**	665,873	66.52	24.36	13.66
**Iohexol**	140,790	18.05	1.73	1.16
**Human brain (7 pool)**	259,200	90,719.04	1.60	1.20

Supplementary Information Table 2. A comparison between the in-vitro CEST-MRF reconstruction results obtained for L-arginine phantoms using the proposed NN and the reference ground truth.

	[L-arg] (mM)		k_{sw} (s^{-1})	
	Neural network	Ground truth reference	Neural network	Ground truth reference
Vial #1	67.22 ± 7.84	64.28 ± 9.34	604.45 ± 122.67	610.42 ± 105.43
Vial #2	58.2 ± 6.87	57.39 ± 6.99	1032.6 ± 57.6	1071.53 ± 74.06
Vial #3	56.9 ± 12.65	54.19 ± 9.94	395.47 ± 144.75	393.19 ± 128.52
